# Serum levels of prostate specific antigen (PSA) after primary vaccination with BNT162b2

**DOI:** 10.1515/almed-2023-0076

**Published:** 2023-08-14

**Authors:** Simone De Nitto, Laura Pighi, Gian Luca Salvagno, Giuseppe Lippi

**Affiliations:** Service of Laboratory Medicine, Pederzoli Hospital, Peschiera del Garda, Italy; Section of Clinical Biochemistry and School of Medicine, University of Verona, Verona, Italy

**Keywords:** COVID-19, prostate, PSA, SARS-CoV-2

**Figure 1:**
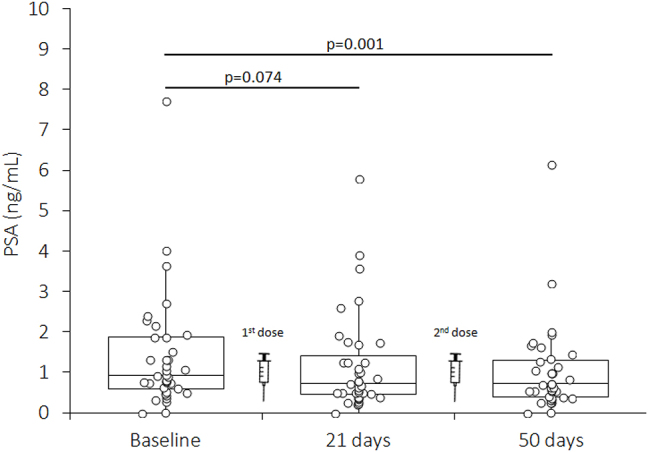
Variation of serum prostate specific antigen (PSA) in ostensibly healthy recipients of a primary cycle of BNT162b2 vaccine. PSA, prostate specific antigen*.*

To the Editor,

Recent evidence suggests that serum levels of prostate-specific antigen (PSA) may increase significantly from baseline in recipients of the Coronavirus Disease 2019 (COVID-19) vaccine BNT162b2 (Pfizer-BioNTech, Mainz, Germany) [1]. To gain further insight into this intriguing topic, we performed a retrospective analysis with 37 male healthy employees (mean age: 61 ± 8 years) of the Pederzoli Hospital in Peschiera del Garda (Verona, Italy) who had completed a primary vaccination cycle with the COVID-19 vaccine BNT162b2. Blood samples were collected by standard venipuncture immediately before the first BNT162b2 dose, 21 days thereafter and thus immediately before the second BNT162b2 dose, and 1 month after the second vaccine dose (i.e., 50 days after the first BNT162b2 dose), as summarized in Figure 1. The full protocol of this study is reported elsewhere [2]. Serum PSA was assayed on a Roche Cobas e801 (Roche Diagnostics, Basel, Switzerland; reportable measuring range: 0.006–100 ng/mL; total imprecision: 1.5–5.1 %). Results of measurements at the three time points were reported as median and interquartile range and compared using paired Mann-Whitney U test (Analyse-it Software Ltd, Leeds, UK). All subjects provided written informed consents for participating to this study, which was conducted in accordance with the Declaration of Helsinki and approved by the Ethics Committee of Verona and Rovigo Provinces (59COVIDCESC; November 8, 2021).

The results of this study are shown in Figure 1. Serum PSA levels were not significantly different from baseline (median: 0.9 ng/mL; IQR: 0.61–1.88 ng/mL) after the first BNT162b2 dose (median: 0.7 ng/mL; IQR: 0.48–1.26 ng/mL; p=0.074), and were even lower than at baseline after the second BNT162b2 dose (median: 0.7 ng/mL; IQR: 0.39–1.29 ng/mL; p=0.001).

Overall, our results do not confirm previous evidence that serum PSA levels may increase after administration of a primary cycle of BNT162b2 vaccine. On the contrary, we found that BNT162b2 vaccine may also have a beneficial effect on prostate biology and function by reducing circulating PSA levels. In particular, we doubt that the PSA change observed by Frumer et al. [1] should really be considered clinically significant. Indeed, the authors reported an increase of 0.03 (IQR: −0.12 to 0.28) and 0.09 (IQR: −0.05 to 0.34) ng/dL after one and three doses of vaccine, respectively. Both values are of low clinical significance, as the median critical difference of this biomarker is 20.5 % [3], orders of magnitude higher than the change reported by Frumer et al. [1].
